# Contracting COVID-19: a longitudinal investigation of the impact of beliefs and knowledge

**DOI:** 10.1038/s41598-021-99981-8

**Published:** 2021-10-14

**Authors:** Courtney A. Moore, Benjamin C. Ruisch, Javier A. Granados Samayoa, Shelby T. Boggs, Jesse T. Ladanyi, Russell H. Fazio

**Affiliations:** 1 Department of Psychology, The Ohio State University, Columbus, OH USA; 2 Institute of Psychology, Leiden University, 2300 RB Leiden, The Netherlands

**Keywords:** Risk factors, Human behaviour

## Abstract

Recent work has found that an individual’s beliefs and personal characteristics can impact perceptions of and responses to the COVID-19 pandemic. Certain individuals—such as those who are politically conservative or who endorse conspiracy theories—are less likely to engage in preventative behaviors like social distancing. The current research aims to address whether these individual differences not only affect people’s reactions to the pandemic, but also their actual likelihood of contracting COVID-19. In the early months of the pandemic, U.S. participants responded to a variety of individual difference measures as well as questions specific to the pandemic itself. Four months later, 2120 of these participants responded with whether they had contracted COVID-19. Nearly all of our included individual difference measures significantly predicted whether a person reported testing positive for the virus in this four-month period. Additional analyses revealed that all of these relationships were primarily mediated by whether participants held accurate knowledge about COVID-19. These findings offer useful insights for developing more effective interventions aimed at slowing the spread of both COVID-19 and future diseases. Moreover, some findings offer critical tests of the validity of such theoretical frameworks as those concerning conspiratorial ideation and disgust sensitivity within a real-world context.

## Introduction

One year after the beginning of the COVID-19 pandemic, more than 100 million individuals worldwide had been infected while the total number of COVID-related deaths surpassed two million^1^. The toll of the virus has been particularly pronounced in the United States, which accounted for approximately one-quarter of these cases and deaths^1,2^. While the recent arrival of multiple COVID-19 vaccines brings hope for curbing the virus, rollout of these vaccines has proved slower than anticipated^3,4^.

Until a more substantial portion of the population is vaccinated, the primary means by which to limit the spread of coronavirus is by following public health guidelines to socially distance, wear a mask while near others, and avoid unnecessary trips outside of the home^5–7^. Many recent studies have focused on the efficacy of these behaviors in preventing the spread of COVID-19. Studies on social distancing have found that communities with greater rates of distancing have lower virus transmission rates^6,8^. Providing convergent support for the effectiveness of distancing, other work has shown that individuals who personally distance more are less likely to contract the virus^9^. Mask use has also proven effective at reducing the transmission of coronavirus, provided the masks are constructed in line with recommendations of the U.S. Centers for Disease Control and Prevention (CDC) or World Health Organization (WHO)^7^.

Despite the wealth of scientific evidence attesting to their efficacy, public health pleas to comply with these preventative behaviors have been met with a variety of responses^10–12^. While some people reliably engage in these preventative behaviors, others overtly oppose the guidelines. Even as the number of COVID-19 cases and deaths reached critical levels, the U.S. witnessed continued protests condemning stay-at-home orders and mask mandates^2,13^.

Scientific research examining the psychological factors that shape a person’s response to the virus has identified several individual differences that predict less concern about the virus and less engagement in preventative behaviors, including greater political conservatism, endorsement of conspiracy theories, and valuing material self-interest or individual freedom over public safety^10,12,14–16^. Although this research has been informative regarding the possible sources of variability in people’s responses to the virus, an important question remains: Do these factors prospectively predict *whether an individual actually contracts COVID-19 over time*? To our knowledge, this is the first study to assess the predictive power of individual differences in beliefs and personal characteristics with respect to the likelihood of contracting the virus. To address this question, we conducted a longitudinal study involving 2120 participants. At Time 1, shortly after the pandemic became a heightened concern for most Americans (Spring 2020), we assessed a wide variety of relevant beliefs, personality characteristics, and demographic factors (e.g., perceptions of the pandemic, trust in scientists, objective knowledge about COVID-19, political ideology). We then followed up with these same participants four months later to assess whether they had contracted the novel coronavirus in the intervening time period. Using these data, we examine which individual differences predict subsequent illness and which do not.

The Time 1 portion of the study had been conducted to test a general theoretical framework regarding compliance with a directive, in this case, the recommendation to practice social distancing^10^. The guiding framework focused on three key components postulated to influence whether or not an individual will comply with any given behavioral directive: perceptions of the *source* of the directive, perceptions of the *challenge* that prompted the directive, and relevant *personal characteristics* of the target.

Regarding the first of these three broad components, the source(s) of the directive, this research identified public health officials and government officials as the two primary sources of the COVID-19 preventative behavior guidelines. As such, participants were asked to indicate their attitudes toward scientists, as perceptions of scientists were expected to directly impact one’s willingness to comply with science-based directives. Participants also reported trust in then-President Trump and the federal government as a whole. This distinction was made given that throughout the pandemic, government officials—including President Trump—have offered contradicting messages about the seriousness of COVID-19 and the need to engage in preventative behaviors^17–21^.

The second component of the compliance framework is perceptions of the challenge itself—in this case, the COVID-19 pandemic. The critical variables within this component were individuals’ perceptions of COVID-19’s seriousness and perceptions of the directives to engage in preventative behaviors (e.g., their efficacy). In addition to subjective perceptions of the pandemic, Fazio and colleagues^10^ also included a measure of objective knowledge of the virus, anticipating that endorsing less correct information—or more *mis*information—about COVID-19 would predict less compliance with the directive.

The third and final component concerns relevant characteristics of the targets of the directive themselves that may influence receptivity. Perhaps unsurprisingly, given the salience, severity, and politicization of the pandemic, this research identified a wide range of relevant characteristics. First, the original study assessed participants’ personal sensitivity to disgust as well as their perceived vulnerability to disease in general^10^. Since the pandemic has been widely politicized^19^, participants also reported their political orientation and which popular news sources they use. Conspiracy theories have also grown in popularity as is common during times of social distress and uncertainty^22,23^. As such, participants reported endorsement of a variety of types of conspiracy theories, with the purpose being to assess whether greater endorsement of conspiracy theories might lead to less support for public health messaging.

Fazio et al.^10^ focused specifically on the relationship between these various predictor variables and social distancing, using both self-reported and “virtual” (i.e., interactive graphical) social-distancing measures. As predicted, all of the previously discussed variables did in fact significantly relate to these measures of social distancing. For example, disgust sensitivity and objective knowledge of COVID-19 were positively associated with greater social distancing. Meanwhile, confidence in President Trump and greater endorsement of conspiracy theories negatively related to distancing behavior. Moreover, a later longitudinal study revealed that participants’ scores on the virtual measure of social distancing behavior were predictive of the likelihood of their subsequently contracting COVID-19^9^.

However, there are several reasons to question whether and to what degree each of these individual difference factors would translate into a higher likelihood of actually contracting the virus. For example, Fazio et al.^9^ focused exclusively on social distancing behavior and did not examine any other preventative behaviors that have been shown to reduce disease transmission, such as hand washing and mask-wearing. Further, several of these associations, although statistically robust, were quite small, and it may be that they are not sufficient to have real-world impacts on disease transmission. Thus, the critical question—whether and to what degree these various source, context, and target factors predict actual COVID-19 contraction—remains unanswered. We address this question in the present research.

In the current study, we followed up with participants from the original research four months later to assess whether they contracted COVID-19. This allowed us to assess which beliefs, personality characteristics, and demographic factors prospectively predicted contracting the virus during the intervening period of time, as well as the relative magnitude of each of these effects. In doing so, we are also able to test critical theoretical questions regarding each of these diverse factors—many of which are theorized to predict illness. For example, past work predicts that individuals who are more sensitive to disgust will be less likely to contract a given illness, as disgust (at least in theory) motivates avoidance of potential pathogens in the environment^24^. In addition, theoretical models of conspiratorial ideation suggest that people who are prone to conspiracy beliefs are likely to be relatively dismissive of anxiety-provoking events such as the current pandemic, as they adopt conspiracy theories as means by which to gain a sense of control over these kinds of situations^25^. As a result, individuals prone to conspiratorial ideation should be more likely to contract the virus. The current study offers an opportunity to test such predictions in a real-world context.

## Results

### Descriptive data

Of the 516 participants (24.3% of the total sample of 2120) who reported having been tested for COVID-19, 116 (5.5% of the total sample) indicated that the test result was positive. Participants who had not been tested were asked whether they nevertheless believed they had contracted the coronavirus. Two-hundred and thirty-two participants (10.9% of the total sample) responded affirmatively. Thus, a total of 348 participants (16.42%) reported having experienced COVID-19 illness at the time of the follow-up survey.

### Predicting a positive COVID-19 test at follow-up

One might argue that individuals with certain beliefs or characteristics were for some reason more or less likely to interpret any ambiguous physical symptoms as an indication of having the virus. In contrast, testing positively for COVID-19 is considerably less ambiguous; it is a clear and salient event that seems relatively unlikely to be mis-construed or mis-reported. As such, we will be featuring analyses in which the outcome is simply whether one tested positively for the virus, versus tested negatively or not being tested at all. However, the results are very similar between this set of analyses and one including our more subjective measure of COVID-19 illness. Results from this more lenient test of our hypotheses are available in section A of the Supplementary Information.

Our major goal is to examine whether each of our variables of interest *prospectively predicts* subsequent illness. Hence, we excluded from the analyses any participants who reported having tested positive for COVID-19 at the time of the initial study. The resulting sample involved 85 participants who subsequently tested positively (coded as 1) and 1993 who reported either a negative test or not having been tested at all (coded as 0). However, the sample size available for any given variable varies as a consequence of the planned missing design employed in the initial studies. That is, because some variables were randomly assigned only to specific subsamples of participants, our analyses for these variables are limited to the participants who completed those measures. Accordingly, in the tables that follow, we also include sample size details for each predictor variable.

To determine which individual difference factors predicted contracting COVID-19, we conducted a series of binary logistic regression analyses examining the dichotomous COVID-19 status variable at follow-up (i.e., tested positive for COVID-19 versus tested negative or not tested at all) as a function of each of the predictor variables. This analysis allowed us not only to assess which variables predicted contracting COVID-19, but also the effect size for each prediction offered by the odds ratio—that is, how the odds of contracting COVID-19 change as a function of a unit change in the predictor variable. The results are summarized in Table 1, which presents, for each variable, the number of participants who did versus did not report having testing positively for the virus and the regression statistics. (To ease interpretation, all continuous predictor variables were standardized).

**Table 1:** Predicting positive COVID-19 test^a^.

	*n*_*1*_/*n*_*2*_	*B*	Wald	*p*	Odds ratio
**Beliefs about the source**					
Trust in scientists^b^	20/542	− 1.043	21.314	0.000	0.352
Trust President Trump re COVID-19 crisis^b^	20/541	0.703	9.682	0.002	2.020
Confidence in federal gov’t effectiveness^b^	20/541	0.997	15.185	0.000	2.709
General confidence in President Trump^b^	20/541	0.551	6.329	0.012	1.735
**Beliefs about the context**					
Worry about contracting virus^b^	85/1993	0.941	51.581	0.000	2.562
Likely to contract virus^b^	85/1993	1.273	86.887	0.000	3.571
Threat (not) exaggerated^b^	85/1993	− 0.737	47.469	0.000	0.478
COVID knowledge^b^	85/1993	− 1.075	195.486	0.000	0.341
Acceptance of rrue items^b^	85/1993	− 0.672	117.127	0.000	0.510
Rejection of false items^b^	85/1993	− 1.023	178.258	0.000	0.360
**Other receptivity-related characteristics**					
General interpersonal compassion^b^	22/571	− 0.462	5.193	0.023	0.630
Disgust sensitivity^b^	29/572	0.936	18.910	0.000	2.549
Perceived vulnerability to disease^b^	29/573	0.201	1.119	0.290	1.223
Preexisting conditions^b^	85/1991	0.670	42.966	0.000	1.953
Political ideology (higher, more conservative) ^b^	85/1992	0.156	2.061	0.151	1.169
Belief in conspiracy theories^b^	29/550	1.448	30.540	0.000	4.253
Science literacy^b^	5/299	− 0.632	2.292	0.130	0.531
Fox News ^c^	57/1087	0.242	1.811	0.178	1.273
NPR ^c^	57/1087	− 1.384	8.048	0.005	0.251
Papers, magazines ^c^	57/1087	− 0.513	6.467	0.011	0.599
**Other target characteristics**					
Age	84/1993	− 0.053	20.980	0.000	0.948
Gender (1 = male/0 = female)	84/1979	0.872	12.888	0.000	2.391
Race (1 = Black/0 = white)	79/1709	1.668	46.312	0.000	5.299

^a^Coded 0 = Negative Test Result or Untested (*n*_*2*_), 1 = Positive Test Result (*n*_*1*_).

^b^Standardized.

^c^Coded 0 = neither watch last week, nor primary news source, 1 = watched last week or primary, 2 = both.

We first examined our broad group of predictor variables that concerned perceptions of the source of the directive, all four of which significantly predicted whether an individual contracted COVID-19. Greater trust in scientists, consistent with our predictions, was associated with reduced likelihood of contracting the virus. Conversely, both greater trust and confidence in President Trump were predictive of an increased likelihood of contracting the virus. Greater trust in the federal government also predicted a greater likelihood of contracting the virus, perhaps due to Trump’s strong association with the federal government (the correlation between each of the measures regarding Trump and that concerning the federal government was *r* = 0.71).

We next turned to factors concerning people’s perceptions of the challenge itself, which also predicted whether an individual tested positive for COVID-19. In particular, the more participants perceived that the threat posed by the pandemic had been exaggerated, the more likely they were to contract the coronavirus. Interestingly, participants also displayed striking insight regarding their personal risks of contracting COVID-19. Those who expressed greater worry about contracting COVID-19, and those who thought they were more likely to contract it, were, in fact, more likely to actually contract the virus. Regarding objective knowledge, having less accurate knowledge about the virus predicted a greater likelihood of subsequently contracting COVID-19. This relation was evident both for the rejection of true statements and the endorsement of misinformation.

Finally, we turned to the third broad group of factors: relevant traits and characteristics of the targets themselves. Interestingly, greater interpersonal compassion was associated with a decreased risk of contracting COVID-19. Conversely, having greater general conspiratorial ideation was significantly associated with an increased likelihood of contracting the virus as was reportedly having pre-existing conditions that increased one’s vulnerability to COVID-19. Finally, an individual’s preferred news sources also appeared to have implications for contracting the virus. Most notably, participants who reported using NPR and/or national newspapers and magazines were less likely to contract COVID-19.

Although the receptivity-related characteristics listed in Table 1 generally showed the expected relations with contracting COVID-19, there was one notable exception: disgust sensitivity. That is, past research and theory posit that greater disgust sensitivity serves to minimize an individual’s contact with potentially pathogenic substances, and thereby reduce one’s likelihood of illness^26,27^. In direct contrast to these predictions, however, individuals who were more sensitive to disgust were *more* likely to contract COVID-19.

With respect to the demographic variables, the findings for a contrast regarding race warrant highlighting. Black participants were approximately five times more likely to report testing positive for COVID-19 than white participants, consistent with past research^28,29^. Younger participants were also more likely to contract the virus, as were males.

Finally, it is worth noting that all the regression outcomes listed in Table 1 remained essentially unchanged when the binary logistic analyses were conducted controlling for annual family income and employment status.

### The mediating role of COVID-specific predictors

Next, we sought to gain insight into *why and how* these various beliefs, personality characteristics, and demographic factors influence a person’s likelihood of contracting COVID-19. Within the current study, predictors addressing one’s beliefs about the context would have developed as the pandemic unfolded (e.g., perceived severity of the threat, knowledge about COVID-19). Meanwhile, other variables we use to predict illness are largely individual differences which presumably would have characterized our participants prior to the pandemic (e.g., political ideology, disgust sensitivity, demographic factors). Consistent with the logic we laid out above, we anticipated that many of these pre-existing individual differences would influence disease contraction indirectly via intermediary perceptions and beliefs about the pandemic.

All our variables addressing perceptions of the pandemic proved to be strong predictors of testing positively for COVID-19 (see Table 1). These were COVID-19 knowledge, believing the threat of COVID-19 to (not) be exaggerated, the degree to which one is worried about personally contracting the virus, and the perceived likelihood of contracting the virus. Given that the latter two were strongly correlated (r = 0.70), each was standardized and then averaged together to form a composite measure we refer to as participants’ perceived risk of contracting COVID-19. Overall, these three variables directly concern people’s perceptions of the pandemic, and as such represent three potential paths by which a given individual difference might relate to one’s risk of contracting COVID-19. Specifically, we hypothesize that our individual difference measures are indirectly predicting illness by leading people to (a) develop less accurate (or more inaccurate) knowledge about COVID-19, (b) minimize the overall threat of the pandemic, and/or (c) accurately perceive that they have a greater risk of contracting the virus.

**Figure 1:**
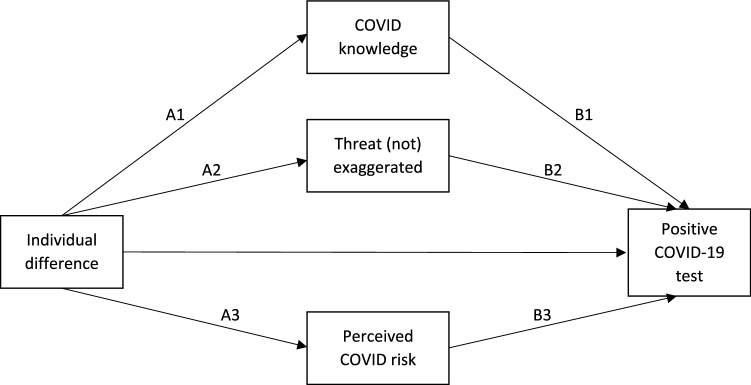
Basic structure of mediational models predicting impact of individual difference variables on positive COVID-19 tests via COVID-specific variables.

Using the PROCESS macro for SPSS (model 4)^30^, we performed a series of mediational analyses examining the relation between each of our individual difference variables and testing positive for COVID-19, mediated by each of the three COVID-specific variables. By considering the mediational variables simultaneously, we assessed the unique variance attributable to each. That is, we assessed the extent to which the relationship between a given individual difference and COVID-19 illness is uniquely mediated by accurate knowledge regarding COVID-19, assessment of the threat posed by the pandemic, and/or perceived risk of contracting the virus. Of course, mediation analyses cannot provide decisive evidence of causal process. Nevertheless, in identifying the factors that statistically account for the relations between our predictor variables and contracting the virus, they can provide some tentative insight into the process by which each of these factors affects probability of disease contraction. Figure 1 offers the standard structure for these mediational models.

The results are summarized in Table 2, in which we present standardized beta coefficients for each individual difference variable as it relates to the COVID-specific variables (Fig. 1 paths A1, A2, and A3). We also include estimates of the overall indirect effects of these individual differences on illness via each of the three mediators (Fig. 1 paths A1*B1, A2*B2, and A3*B3). These estimates—which are themselves the products of the respective A and B paths (e.g., A1*B1)—allow us to assess the extent to which each of these COVID-specific variables accounts for the relationships we uncovered between our individual difference measures and COVID-19 illness. Full mediational models for each individual difference variable are included in section B of the Supplementary Information.

**Table 2:** Indirect effects of individual difference variables on testing positive for COVID-19 via COVID-specific variables.

	COVID knowledge		Threat (not) exaggerated		Perceived COVID risk	
	Standardized beta (A1)	Indirect effect (A1*B1)	Standardized beta (A2)	Indirect effect (A2*B2)	Standardized beta (A3)	Indirect effect (A3*B3)
**Beliefs about the source**						
Trust in scientists	0.581***	− 0.379^†^	0.552***	− 0.116	0.059	0.058
Trust President Trump re COVID-19 crisis	− 0.486***	0.413^†^	− 0.538***	0.173	− 0.117**	− 0.112^†^
Confidence in federal gov’t effectiveness	− 0.367***	0.236^†^	− 0.340***	0.061	− 0.059	− 0.058
General confidence in President Trump	− 0.460***	0.410^†^	− 0.536***	0.206	− 0.128**	− 0.126^†^
**Other receptivity-related characteristics**						
General interpersonal compassion	0.196***	− 0.163^†^	0.219***	− 0.015	0.074	0.013
Disgust sensitivity	− 0.320***	0.306^†^	0.082	0.001	0.271***	0.321^†^
Perceived vulnerability to disease	0.030	− 0.028	0.196***	0.002	0.419***	0.464^†^
Preexisting conditions	− 0.203***	0.165^†^	0.051*	− 0.010	0.364***	0.287^†^
Political ideology (higher, more conservative)	− 0.263***	0.224^†^	− 0.413***	0.087	− 0.135***	− 0.106^†^
Belief in conspiracy theories	− 0.509***	0.353^†^	0.265***	0.127	0.102*	0.102^†^
Science literacy^a^	0.434***	− 0.310	− 0.159**	− 0.073	− 0.121*	− 0.212
Fox News^b^	− 0.273***	0.284^†^	− 0.364***	0.114	− 0.030	− 0.028
NPR^b^	0.352***	− 0.348^†^	0.350***	− 0.106	0.138**	0.134^†^
Papers, magazines^b^	0.213***	− 0.218^†^	0.210***	− 0.061	0.089*	0.083^†^
**Other target characteristics**						
Age	0.073***	− 0.051^†^	− 0.006	0.002	− 0.025	− 0.022
Gender (1 = male/0 = female)	− 0.246***	0.198^†^	− 0.207***	0.036	− 0.125**	− 0.102^†^
Race (1 = Black/0 = white)	− 1.052***	0.916^†^	0.069	0.010	0.355***	0.247^†^

^a^ Statistics for this variable should be interpreted cautiously as the bootstrapped mediational model failed to converge, presumably due to the small number of positive test cases (5 out of 304).

^b^ Coded 0 = neither watch last week, nor primary news source, 1 = watched last week or primary, 2 = both.

*p < .05; **p < .01; ***p < .001.

^†^ Denotes a 95% bias-corrected bootstrap confidence interval (using 10,000 bootstrap samples) that does not include zero.

Interestingly, several of our individual difference measures differentially relate to the COVID-specific variables. When the three COVID-specific variables are entered together as potential mediators for the relationships between each of the four source variables and COVID illness, all four relationships appear to be mediated by COVID knowledge. This suggests that positive assessments of Trump and the federal government may have led people to develop less knowledge about the virus, while trust in science led people to develop greater knowledge. These knowledge differences, in turn, appear as though they may have prompted people to act in ways that affected their probability of contracting the virus.

The two variables specific to Trump (trust in Trump to lead us through the pandemic, and general confidence in Trump) were additionally mediated to a lesser degree by individuals’ insights regarding their perceived risk of contracting the virus. Interestingly, trust in Trump was associated with lower perceptions of one’s personal risk of contracting the virus. Although the direct relationship between these Trump-relevant variables and COVID illness is positive (i.e., more trust or confidence in Trump predicts greater likelihood of illness), the overall indirect effect of these Trump-relevant variables on illness via perceived risk of COVID-19 is negative. That is, greater confidence in Trump was associated with a decrease in perceived risk of COVID-19, and such individuals were in fact less likely to contract the virus.

Turning to the receptivity-related characteristics, we find that COVID knowledge acted as a strong mediator for nearly all the variables. The relation between general compassion and illness was mediated by the development of more accurate knowledge, whereas the relations for disgust sensitivity, political conservatism, and conspiratorial ideation were mediated by less accurate knowledge. The relations with the various news sources also were mediated largely by the COVID knowledge variable, with Fox News being associated with less accuracy, and NPR and national newspapers and magazines with more accurate COVID information.

Insight into one’s perceived risk of contracting COVID-19 emerged as the primary mediator for the relations involving perceived vulnerability to disease and pre-existing conditions. Some of the other receptivity variables were also mediated by perceived risk, although generally to a lesser extent than the mediational role of COVID knowledge. Greater disgust sensitivity and greater conspiratorial ideation involved an accurate sense that one was more at risk, whereas more conservative individuals tended to perceive themselves to be at a lower risk of contracting COVID-19 (parallel to the findings for the Trump-relevant source variables). Finally, followers of NPR and national newspapers/magazines were also more likely to consider themselves at a higher risk of contracting COVID-19, and this insight proved correct.

Lastly, we find that the relations of age, gender, and the Black/white race variable with testing positively for COVID-19 were all mediated primarily by their associations with the development of accurate knowledge. Younger individuals, males, and Black participants were likely to have less accurate knowledge about COVID-19 and were more likely to contract the virus. Gender and the race variable were also mediated, to a lesser extent, by perceptions of risk. Female and Black participants who perceive themselves to be at a higher risk are indeed more likely to have contracted COVID-19.

In summary, when accounting for potential mediating effects of the three COVID-specific variables simultaneously, COVID knowledge emerged as the primary mediator for the effects of our individual differences on COVID illness. Furthermore, we find that while additional variance in several of our mediational models was accounted for by participants’ assessments of their personal risk of contracting COVID-19, perceptions of the threat posed by the pandemic did not show a mediational effect for any of our predictors.

## Discussion

This research demonstrates the importance of an individual’s beliefs and personal characteristics for predicting a critically important health outcome—whether said individual is likely to contract COVID-19. We identified several powerful predictors of contracting illness, including trust in the major sources of information about COVID-19 (e.g., scientists, the president), beliefs about the severity of the pandemic itself, personal insights about contracting the virus, and accurate knowledge about COVID-19. As such, these findings could be leveraged to reduce the spread of the COVID-19 virus. For example, many of our findings point to the politicized nature of the pandemic in the U.S. As many conservatives—including former President Trump—largely dismissed the significance of COVID-19 and/or occasionally conveyed misinformation about the virus, individuals on the political right are less likely to report engaging in preventative measures such as social distancing^10,11,31^. Our research demonstrates that one’s political beliefs (in particular, trust in President Trump and the federal government) not only affect self-reported attitudes or beliefs, but one’s actual health. Thus, it is imperative that government and public health officials aim to reduce this political divide and avoid further politicization of this and any future pandemics. Our data suggests a few paths by which to do so: by promoting the dissemination of accurate knowledge, dispelling misinformation, and undercutting conspiracy theories.

In addition, many theoretically relevant individual characteristics were predictive of subsequent illness. Especially noteworthy were the associations involving conspiratorial ideation—which supported dominant theoretical models—and disgust sensitivity—which contradicted past research and theory. As noted earlier, belief in conspiracy theories has been shown to relate to a number of important political and health outcomes^25,32^. However, the present findings illustrate the significance of general conspiratorial ideation in that the measure involved not any conspiracy theories that directly concerned COVID-19, but generic beliefs regarding such matters as alien contact and powerful, secretive forces^33^. Past work has found systematic increases in conspiracy beliefs during times of turmoil, with some positing that conspiracy theories seem attractive during these times as they offer explanations that allow individuals to maintain a particular worldview^22,23^. This explanation argues that conspiracy theories may act as a buffer against stress or uncertainty. In doing so, though, these individuals may come to not only downplay the significance of the existing threat, but also develop less accurate knowledge of the threat. Our findings support this theoretical explanation, as conspiratorial ideation was associated strongly with less accurate knowledge regarding COVID-19—a factor which, in turn, increased the likelihood of contracting COVID-19. To our knowledge, this is the first demonstration that conspiracy beliefs prospectively predict a negative health outcome while also providing critical information regarding how these effects unfold.

Also of particular interest are the findings related to disgust sensitivity, which appeared to run directly counter to theoretical models of disgust. Greater disgust sensitivity was associated not with a reduced likelihood of contracting the virus, but rather with an increased likelihood of doing so. The direction of this relation poses a serious challenge to theoretical frameworks that view disgust sensitivity as a disease-avoidance mechanism^34–36^. Our mediational analyses showed that the positive relationship between disgust sensitivity and contracting COVID-19 was mediated by both COVID knowledge and perceived COVID-19 risk, with disgust sensitivity being negatively related to COVID knowledge and positively related to assessments of personal risk. The latter may reflect a propensity for individuals characterized by greater disgust sensitivity to be more insightful regarding their actual risk of contracting the virus, similar to those who report preexisting conditions or general vulnerability to disease.

An additional possibility is suggested by the observed mediational role of COVID-19 knowledge. Greater disgust sensitivity was associated with less accurate knowledge, which was itself predictive of a greater likelihood of testing positively for the virus. It may be that individuals characterized by more disgust sensitivity tended to avoid and/or not fully process the validity of information regarding the virus, possibly because they experienced disgust reactions in response to such information. Given the critical role knowledge appears to play, any such avoidance may have countered the postulated disease avoidance function of disgust sensitivity.

To further evaluate this possibility with the current data, we ran a binary logistic regression model with both disgust sensitivity and COVID-19 knowledge as predictors of illness. Within this model, COVID-19 knowledge was a significant predictor of reports of having tested positively (*β* = –1.06, *SE* = 0.15, Wald = 50.40, *p* = 0.000), whereas disgust sensitivity was not (*β* = 0.36, *SE* = 0.26, Wald = 1.95*, p* = 0.162). In other words, COVID-19 knowledge appears to attenuate the unexpected effect of disgust sensitivity on likelihood of contracting the virus. Future research will need to further address why disgust sensitivity did not have the postulated beneficial effects on avoiding COVID-19, and whether this calls into question the general theoretical premise regarding disgust sensitivity and disease avoidance, or whether the absence of any signs of a positive relation might be specific to other features of the COVID-19 pandemic.

Finally, our second set of analyses (Table 2) aimed to further understanding of the relationships between our individual difference variables and COVID illness by examining the potential mediational roles of variables specific to the pandemic: COVID knowledge, assessments of the threat posed by the pandemic, and personal risk of contracting COVID-19. While we saw in the initial set of analyses (Table 1) that accurate knowledge about COVID-19 was a strong predictor of illness, our mediational analyses reveal it to be a mechanism by which many pre-existing individual differences come to impact likelihood of contracting COVID-19. In fact, when controlling for the potential mediational effects of other COVID-specific variables (i.e., perception of the threat of the pandemic, personal insight about COVID-19), COVID knowledge was identified as the primary pathway relating our individual differences to the likelihood of testing positive for COVID-19. These mediational analyses suggest that increasing accurate COVID knowledge—and decreasing endorsement of misinformation—should reduce the negative impact of individual differences such as conspiratorial ideation and lack of trust in scientists on likelihood of contracting the virus. As such, COVID knowledge could be a critical target for interventions aimed at decreasing the spread of COVID-19.

What is not yet known is exactly why certain individuals develop greater COVID knowledge. For example, the relationship between trust in scientists and testing positive for COVID-19 was mediated by COVID-19 knowledge in that those who trust scientists had higher rates of accurate knowledge, which decreased their likelihood of illness. It could be that individuals who are inclined to trust scientists purposely sought out more accurate information, that they tend to be in environments with better access to scientifically rigorous information, and/or that they approached misinformation with greater skepticism. Whatever the specific means, past work has shown that individuals with higher rates of knowledge are more likely to follow preventative measures^10,37^, which in turn makes them less likely to contract COVID-19^7,9,38^. While our data offers some unique support for this theoretical reasoning, future research would need to evaluate in a progressive, longitudinal manner the full path from individual difference variables to knowledge acquisition to voluntary preventative behaviors and, ultimately, illness.

Our mediational analyses also offer insight into findings relating political beliefs and identity to COVID illness. Although we found substantial relationships between illness and specific political beliefs such as trust in Trump and confidence in the federal government, we observed only a nonsignificant trend associating self-reported political conservatism with a greater likelihood of having tested positively for COVID-19. This is somewhat surprising given that other recent work has found that U.S. counties with higher rates of conservatism (as indexed by the proportion of votes for Trump versus Clinton in the 2016 election) have higher rates of COVID-19 illness^39^. In a parallel fashion, considerable research has documented that political conservatives are less likely than liberals to pursue various preventative behaviors, such as social distancing^10–12,17,39^. Our mediational analysis sheds light on this seeming discrepancy. It revealed divergent paths by which political ideology relates to the likelihood of contracting COVID-19, as indexed by a positive test outcome (versus a negative test or not having been tested at all). As shown in Table 2, political ideology was associated with significant indirect effects via both COVID knowledge and perceived risk of COVID-19. Importantly, these two indirect paths offer opposing predictions for subsequent COVID illness. For one, we find that more conservative individuals are less likely to have accurate COVID knowledge, which is itself related to an increased likelihood of contracting the virus. However, more conservative individuals are also less likely to perceive themselves to be at risk of contracting COVID-19, and we find such risk perceptions to accurately predict likelihood of illness. Risk perceptions are known to be associated with county-level COVID-19 infection and death rates^40^. Hence, the perception of lesser exposure to the virus by the more conservative participants in our sample worked in opposition to their less accurate COVID knowledge with respect to their subsequent likelihood of contracting the virus. As such, our analyses offer a more nuanced picture of the relationship between political ideology and contracting COVID-19. More research is needed to assess why particular individuals may be characterized by either of these two divergent paths over the other, and why specific political beliefs, like trust in Trump, offer greater predictive power than simple political identity within the COVID-19 context. Nevertheless, the mediational analysis revealed a very substantial indirect effect of political ideology on illness through COVID knowledge, which very much parallels our and others’ earlier observations about the politicized nature of the pandemic^10–12,17,39,40^.

In conclusion, the current research offers novel evidence regarding the importance of one’s beliefs and personal characteristics in predicting the likelihood of contracting the COVID-19 virus. The findings identify personal, psychological factors that appear to predispose a person to undue risk of contracting COVID-19. While some of the variables we assessed directly concerned the pandemic (e.g., knowledge about COVID-19, believing the threat of COVID-19 to be exaggerated), many were individual differences that were likely to have characterized the participants prior to the pandemic’s emergence (e.g., conspiratorial ideation, political ideology, interpersonal compassion). Yet, these too had meaningful effects. Moreover, our mediational analyses in particular provide initial evidence as to how and why each of these individual difference factors relate to disease contraction. This work provides unique insights into how public health officials, politicians, health psychologists, and other social scientists might develop more effective messaging and policies—as well as which groups and individuals should be most actively targeted by these campaigns—to curb the transmission of COVID-19 and similar diseases in the future.

## Methods

We recruited our participant samples from Amazon Mechanical Turk^41^. Although not representative of the U.S. population, MTurk samples tend to be more demographically, politically, and geographically diverse than the samples typically used in psychological research^42^. They also perform similarly to non-MTurk samples across many tasks and measures^43,44^, including surveys on social and political attitudes^45^. Most importantly, however, our aim is not to make claims regarding the absolute frequency of COVID-19 illness in the population. Rather, we seek to understand which beliefs and characteristics predict whether an individual contracts the coronavirus over time. Given this aim, MTurk participants offer an appropriate test of our research questions.

Given the large number of variables we measured in these studies, we employed a “planned missing” design^46^ in which different subgroups of participants completed different sets of predictor measures. First, all participants completed a set of measures regarding their beliefs and behaviors concerning the pandemic, as well as various demographic items and relevant covariate measures. We refer to this common set of measures as “the core survey.” Participants were then randomly assigned to one of four subsets of the remaining predictor variables, which were grouped according to theoretical and empirical relatedness. The four sub-studies included: (a) beliefs about the sources of the directive, (b) news sources and endorsement of conspiracy theories, (c) general interpersonal compassion, and (d) disgust sensitivity and perceived vulnerability to disease.

### Participants

The sample consisted of MTurk workers who had participated in one of two studies conducted in Spring 2020. All participants who had granted permission to re-contact them were invited to complete a brief survey approximately four months after their initial study for a payment of $1. A total of 2120 individuals, all US residents, completed this survey (1031 women, 1074 men, 15 no response; *M*_age_ = 40.39, *SD*_*age*_ = 15.34). Study 1 was completed on May 7–8 (*n* = 1281; see Fazio et al.^10^ for a detailed report of this study) and Study 2 on June 9 (*n* = 839). This research was reviewed and approved by The Ohio State University’s Office of Responsible Research Practices (IRB). All methods were performed in accordance with the relevant guidelines and regulations, and informed consent was provided by all participants.

### Measures

After providing informed consent, participants completed a wide range of questions regarding the COVID-19 pandemic. These included the critical questions described above concerning their perceptions of the pandemic (e.g., whether it has been exaggerated; worry about contracting the virus), a test assessing knowledge about COVID-19, the subset of predictor variables to which they had been randomly assigned, and a series of demographic questions. In addition, participants also completed a set of measures concerning their behavior regarding the pandemic (e.g., self-reported behaviors, virtual measures of social distancing). Given that the relation between these latter measures of social distancing behavior and contracting COVID-19 were the focus of an earlier report^9^, they are not analyzed here.

#### Perceptions of the pandemic

Participants completed a set of items regarding their perceptions of the pandemic. They were first asked how worried they were about personally contracting the novel coronavirus, how likely they thought they were to contract the virus, and how concerned they were about the spread of the virus in general. The last item asked whether they believed that the threat of COVID-19 had been “greatly exaggerated,” “somewhat exaggerated,” “adequately conveyed,” or “not conveyed strongly enough.”

#### COVID-19 knowledge

This brief test of objective COVID-19 knowledge consisted of 13 statements, all either facts or myths about COVID-19, based on information from the CDC and the WHO. Participants were asked to indicate whether each statement was true or false. Examples of true statements include “Some individuals who have COVID-19/the coronavirus do not show any symptoms” and “Washing one's hands with soap and water for at least 20 s can reduce the spread of COVID-19/the coronavirus.” Meanwhile, false statements included “Antibiotics are an effective treatment for COVID-19/the coronavirus” and “Spraying chlorine on my body will protect me even if COVID-19/the coronavirus has already entered my system.” We summed the number of correct responses to create an index of objective knowledge (α = 0.64). We also computed the correct number of responses for both true and false items separately to independently assess the effects of acceptance of true information versus rejection of falsehoods (α = 0.63 and α = 0.84, respectively).

#### Faith in government

Throughout the pandemic, different—and sometimes contradictory—information has been communicated by various government entities. We asked participants to provide their perceptions of a few of these major entities within four items, each with seven-point response scales ranging from “Not at all” to “Very much.” For example, participants were asked whether they “trust President Trump to lead us effectively through the COVID-19 crisis.” They also rated their general confidence in both President Trump (“Generally speaking, how much confidence do you have in President Trump?”) and the federal government (“Generally speaking, how confident are you that the federal government will address the nation’s problems effectively?”).

#### Trust in scientists

To assess participants’ trust in scientists, we used a shortened version (the 11 items with the highest corrected item-total correlation) of a measure developed by Nadelson et al.^47^. Using a five-point scale ranging from “Strongly disagree” to “Strongly agree,” participants rated statements including “We should trust the work of scientists” and “We cannot trust scientists because they are biased in their perspectives” (reverse coded). We averaged across these items to compute a composite rating (α = 0.81).

#### Science literacy

The Civic Scientific Literacy Scale^48^ was used to assess participants’ general scientific knowledge. As a note, this measure was included in only one of the two initial studies (Study 1, *n* = 1281). Participants indicated whether they agreed or disagreed with 11 statements, including “The Earth goes around the Sun once each year” and “Electrons are smaller than atoms.” The number of correct responses served as an indicator of participants’ general scientific literacy (α = 0.58). While the internal consistency of this measure may be considered low for a general psychological scale, this measure consists of a limited set of statements which fall under the broad topic of science but are not inherently homogenous. As various psychometricians have noted, such scales are likely to have relatively low values for Cronbach’s alpha even though the items still fall within the same dimension or category (e.g., scientific facts)^49,50^. Nevertheless, this brief quiz provides a useful index of individual’s general scientific knowledge.

#### Endorsement of conspiracy theories

Participants’ tendency towards conspiratorial ideation was measured with the Generic Conspiracist Beliefs scale^32^, which assesses endorsement of a variety of generic conspiracy theories. The 15 items of this scale include statements such as “Technology with mind-control capacities is used on people without their knowledge,” “The government permits or perpetrates acts of terrorism on its own soil, disguising its involvement,” and “Evidence of alien contact is being concealed from the public.” Participants rated their endorsement of each statement on a five-point scale ranging from “Definitely not true” to “Definitely true,” and the average rating across the 15 items was computed (α = 0.96).

#### News sources

Participants were asked to select all of the news sources from which they had gotten their news in the past week from the following list: CNN, Fox News, MSNBC, NPR, national newspapers and magazines, social media, and ABC, CBS, or NBC News, as well as the option “do not follow the news.” Participants who selected at least one news source were then asked to indicate which of these sources they considered to be their primary news source. For our purposes, we were especially interested in exposure to Fox News as other work has shown that following Fox News relates strongly to attitudes toward the pandemic^51^.

#### General interpersonal compassion

Fourteen items from a scale developed by Davis^52^ were used to assess participants’ general compassion for others. Statements included “I often have tender, concerned feelings for people less fortunate than me” and “Before criticizing somebody, I try to imagine how I would feel if I were in their place.” Participants responded to each item using a five-point scale ranging from “Does not describe me well” to “Describes me very well.” Ratings were averaged to compute an overall metric of interpersonal compassion (α = 0.87).

#### Disgust sensitivity

We assessed participants’ sensitivity to disgust using the five-item contamination subscale of the Disgust Scale-Revised^26,27^. Three items (e.g., “A friend offers you a piece of chocolate shaped like dog-doo”) were rated using a five-point scale ranging from “Not disgusting at all” to “Extremely disgusting,” while the other three (e.g., “I never let any part of my body touch the toilet seat in a public washroom”) were rated using a five-point scale ranging from “Strongly disagree” to “Strongly agree.” The overall score for disgust sensitivity was computed by averaging across the five items (α = 0.70).

#### Perceived vulnerability to disease

Duncan, Schaller, and Park’s^53^ 15-item scale was used to assess participants’ perceptions of their likelihood of contracting disease or illness in general. Statements included “If an illness is ‘going around’, I will get it” and “It does not make me anxious to be around sick people” (reverse-scored). Participants rated their agreement with each item on a five-point scale from “Strongly disagree” to “Strongly agree.” After recoding the reverse-scored items, the average rating across the 15 items was computed (α = 0.73).

#### Preexisting conditions

Having preexisting health conditions was identified a priori as a likely predictor of contracting COVID-19. As such, participants were asked to consider their “personal health prior to the outbreak of the COVID-19 virus” and to then indicate whether they would have described themselves “as having pre-existing medical conditions that left you more vulnerable to the virus than the average person” by selecting one of five response options ranging from “Definitely not” to “Definitely yes.”

#### Demographics

Other work has shown that certain social groups in the U.S. tend to have higher rates of infection. This includes the elderly who are more likely to contract the virus, as well as to have more complications with the disease. Additionally, systemic and structural factors in US society have led racial and ethnic minorities to have higher infection, hospitalization, and death rates than non-Hispanic white Americans^28,29^. For example, the COVID-19 death rate of Black Americans is reported to be more than twice that of white Americans. Given this, participants were asked to report their age, gender, race and/or ethnicity. Additionally, participants indicated their political ideology on a seven-point scale ranging from “Extremely liberal” to “Extremely conservative.”

#### Follow-up survey

Four months after the initial study, participants completed a brief survey assessing whether they had or had not contracted the coronavirus. They were first asked whether they had been tested for COVID-19. If so, they indicated whether the test showed that they had COVID-19. If they had not been tested, they were asked “Even though you may not have been tested, do you believe that you have ever had COVID-19 / the coronavirus?” to which they responded yes or no. Participants who reported either testing positively or believing they had COVID-19 were then asked to select from a list of possibilities to indicate how they thought they might have contracted the virus.

## Supplementary Information

Supplementary Information.

## Data Availability

The datasets generated and analyzed during the current study are available on the Open Science Framework at https://osf.io/ywv5r/?view_only=6dd2b2715fe349bd8b2252624d25ad3d.
