# Supplementary material for: Contracting COVID-19: a longitudinal investigation of the impact of beliefs and knowledge
Source: Sci Rep. 2021 Oct 14;11:20460. doi: 10.1038/s41598-021-99981-8 (PMC8516850; doi:10.1038/s41598-021-99981-8)
Supplement: Supplementary file 1 — Supplementary Information. [file 41598_2021_99981_MOESM1_ESM.docx]

**Supplementary Information**

1. **Predicting Reports of Having Contracted COVID-19 at Follow-up**

This set of analyses focused on the comparison of those participants who reported not having contracted COVID-19 (coded as 0) to those who reported either having tested positively or believing they had contracted COVID-19 despite having not been tested (coded as 1). Our major interest was to examine whether each of our variables of interest prospectively predicts subsequent illness. Hence, we excluded from the analyses any participants who reported having COVID-19 at the time of the initial study. Of the 2,120 follow-up participants, 235 had either reported a positive test result at Time 1 or reported that they believed they had COVID-19 but had not been tested. These participants were therefore excluded from analyses, resulting in a total sample of 1,885 participants for this set of analyses. However, the sample size available for any given variable varies as a consequence of the planned missing design employed in the initial studies. That is, because some variables were randomly assigned only to specific subsamples of participants, our analyses for these variables are limited to the participants who completed those measures. Accordingly, in the table that follows, we include sample size details for each predictor variable.

To determine which individual difference factors predicted contracting COVID-19, we conducted a series of binary logistic regression analyses examining the dichotomous COVID-19 status variable at follow-up (i.e., did versus did not report contracting COVID-19) as a function of each of the predictor variables. This analysis allowed us not only to assess which variables predicted reports of COVID-19, but also the effect size for each prediction offered by the odds ratio—that is, how the odds of contracting COVID-19 change as a function of a unit change in the predictor variable. The results are summarized in Table 1, which presents, for each variable, the number of participants who did versus did not report having contracted the virus and the regression statistics. (To ease interpretation, all continuous predictor variables were standardized.)

***Supplementary Table A.1. Predicting reports of COVID-19 status^a^***

|  | ***n_1_*/*n_2_*** | ***B*** | **Wald** | ***p*** | **Odds ratio** |
| --- | --- | --- | --- | --- | --- |
| **Beliefs about the source** |  |  |  |  |  |
| Trust in scientists^b^ | 59/452 | -0.356 | 7.109 | 0.008 | 0.701 |
| Trust President Trump re COVID-19 crisis^b^ | 59/451 | 0.359 | 7.334 | 0.007 | 1.432 |
| Confidence in federal gov’t effectiveness^b^ | 59/451 | 0.409 | 8.534 | 0.003 | 1.506 |
| General confidence in President Trump^b^ | 59/451 | 0.282 | 4.503 | 0.034 | 1.326 |
|  |  |  |  |  |  |
| **Beliefs about the context** |  |  |  |  |  |
| Worry about contracting virus^b^ | 199/1686 | 0.409 | 28.121 | 0.000 | 1.506 |
| Likely to contract virus^b^ | 199/1686 | 0.590 | 57.816 | 0.000 | 1.804 |
| Threat (not) exaggerated^b^ | 199/1686 | -0.430 | 35.573 | 0.000 | 0.651 |
| COVID knowledge^b^ | 199/1686 | -0.680 | 141.664 | 0.000 | 0.507 |
| Acceptance of rrue items^b^ | 199/1686 | -0.467 | 75.038 | 0.000 | 0.627 |
| Rejection of false items^b^ | 199/1686 | -0.655 | 127.062 | 0.000 | 0.520 |
|  |  |  |  |  |  |
| **Other receptivity-related characteristics** |  |  |  |  |  |
| General interpersonal compassion^b^ | 49/487 | -0.233 | 2.562 | 0.109 | 0.792 |
| Disgust sensitivity^b^ | 62/485 | 0.512 | 13.386 | 0.000 | 1.669 |
| Perceived vulnerability to disease^b^ | 62/486 | 0.328 | 5.841 | 0.016 | 1.389 |
| Preexisting conditions^b^ | 199/1684 | 0.350 | 25.443 | 0.000 | 1.420 |
| Political ideology (higher, more conservative) ^b^ | 198/1686 | 0.229 | 9.567 | 0.002 | 1.257 |
| Belief in conspiracy theories^b^ | 59/464 | 0.664 | 2.425 | 0.000 | 1.943 |
| Science literacy^b^ | 29/249 | -0.401 | 4.602 | 0.032 | 0.670 |
| Fox News ^c^ | 125/903 | 0.343 | 7.472 | 0.006 | 1.409 |
| NPR ^c^ | 125/903 | -0.851 | 12.005 | 0.001 | 0.427 |
| Papers, magazines ^c^ | 125/903 | -0.363 | 7.436 | 0.006 | 0.695 |
|  |  |  |  |  |  |
| **Other Target Characteristics** |  |  |  |  |  |
| Age | 198/1686 | -0.028 | 17.681 | 0.000 | 0.972 |
| Gender (1=male/0=female) | 198/1674 | 0.157 | 1.085 | 0.298 | 1.170 |
| Race (1=Black/0=white) | 175/1438 | 0.996 | 25.133 | 0.000 | 2.708 |

^a^ Coded 0 = No report of COVID-19 (*n_2_*), 1 = Tested Positively or Untested but believe had COVID-19 (*n_1_*)

^b^ Standardized

^c^ Coded 0=neither watch last week, nor primary news source, 1=watched last week or primary, 2=both

1. Mediational Models

Included here are all relevant statistics for our mediational models (reported partially in Table 2 of the main text). Each figure depicts the model in full with statistics included for all direct paths. Each table includes statistics for indirect effects within the model.

*Supplementary Figure B.1. Direct effects of trust in scientists on COVID-19 test results mediated by COVID-specific predictors*

COVID knowledge

Trust in scientists

Positive COVID-19 test

Threat (not) exaggerated

Perceived COVID risk

0.581***

0.552***

0.059

-0.651*

-0.210

0.977**

-0.189

**p* < 0.05; ***p* < 0.01; ****p* < 0.001

*Supplementary Table B.1. Indirect effects of trust in scientists on COVID-19 test results*

|  | Indirect effect | Standard error | 95% confidence interval |
| --- | --- | --- | --- |
| TOTAL | -0.437^†^ | 0.197 | [-0.886, -0.104] |
| COVID knowledge | -0.379^†^ | 0.206 | [-0.855, -0.036] |
| Threat (not) exaggerated | -0.116 | 0.205 | [-0.506, 0.286] |
| Perceived COVID risk | 0.058 | 0.062 | [-0.031, 0.206] |

^†^Denotes a 95% bias-corrected bootstrap confidence interval (using 10,000 bootstrap samples) that does not include zero

*Supplementary Figure B.2. Direct effects of trust in Trump (re COVID-19 crisis) on COVID-19 test results mediated by COVID-specific predictors*

COVID knowledge

Trust in Trump

Positive COVID-19 test

Threat (not) exaggerated

Perceived COVID risk

-0.486***

-0.538***

-0.117**

-0.850***

-0.321

0.960**

-0.344

**p* < 0.05; ***p* < 0.01; ****p* < 0.001

*Supplementary Table B.2. Indirect effects of trust in Trump (re COVID-19 crisis) on COVID-19 test results*

|  | Indirect effect | Standard error | 95% confidence interval |
| --- | --- | --- | --- |
| TOTAL | -0.474^†^ | 0.219 | [0.062, 0.937] |
| COVID knowledge | 0.413^†^ | 0.157 | [0.163, 0.774] |
| Threat (not) exaggerated | 0.173 | 0.229 | [-0.279, 0.647] |
| Perceived COVID risk | -0.112^†^ | 0.071 | [-0.292, -0.017] |

^†^Denotes a 95% bias-corrected bootstrap confidence interval (using 10,000 bootstrap samples) that does not include zero

*Supplementary Figure B.3. Direct effects of confidence in federal government (re COVID-19 crisis) on COVID-19 test results mediated by COVID-specific predictors*

COVID knowledge

Confidence in federal government

Positive COVID-19 test

Threat (not) exaggerated

Perceived COVID risk

-0.367***

-0.340 ***

-0.059

-0.643*

-0.178

0.987**

0.309

**p* < 0.05; ***p* < 0.01; ****p* < 0.001

*Supplementary Table B.3. Indirect effects of confidence in federal government (re COVID-19 crisis) on COVID-19 test results*

|  | Indirect effect | Standard error | 95% confidence interval |
| --- | --- | --- | --- |
| TOTAL | 0.238 | 0.131 | [-0.016, 0.508] |
| COVID knowledge | 0.236^†^ | 0.111 | [0.049, 0.491] |
| Threat (not) exaggerated | 0.061 | 0.133 | [-0.220, 0.309] |
| Perceived COVID risk | -0.058 | 0.057 | [-0.195, 0.028] |

^†^Denotes a 95% bias-corrected bootstrap confidence interval (using 10,000 bootstrap samples) that does not include zero

*Supplementary Figure B.4. Direct effects of general confidence in Trump on COVID-19 test results mediated by COVID-specific predictors*

COVID knowledge

Confidence in Trump

Positive COVID-19 test

Threat (not) exaggerated

Perceived COVID risk

-0.460***

-0.536***

-0.128**

-0.893***

-0.385

0.986**

-0.543

**p* < 0.05; ***p* < 0.01; ****p* < 0.001

*Supplementary Table B.4. Indirect effects of general confidence in Trump on COVID-19 test results*

|  | Indirect effect | Standard error | 95% confidence interval |
| --- | --- | --- | --- |
| TOTAL | 0.491^†^ | 0.197 | [0.117, 0.910] |
| COVID knowledge | 0.410^†^ | 0.147 | [0.166, 0.747] |
| Threat (not) exaggerated | 0.206 | 0.214 | [-0.208, 0.638] |
| Perceived COVID risk | -0.126^†^ | 0.075 | [-0.316, -0.026] |

^†^Denotes a 95% bias-corrected bootstrap confidence interval (using 10,000 bootstrap samples) that does not include zero

*Supplementary Figure B.5. Direct effects of general interpersonal compassion on COVID-19 test results mediated by COVID-specific predictors*

COVID knowledge

Interpersonal compassion

Positive COVID-19 test

Threat (not) exaggerated

Perceived COVID risk

0.196***

0.219***

0.074

-0.831***

-0.069

0.182

-0.167

**p* < 0.05; ***p* < 0.01; ****p* < 0.001

*Supplementary Table B.5. Indirect effects of general interpersonal compassion on COVID-19 test results*

|  | Indirect effect | Standard error | 95% confidence interval |
| --- | --- | --- | --- |
| TOTAL | -0.165 ^†^ | 0.079 | [-0.335, -0.021] |
| COVID knowledge | -0.163^†^ | 0.049 | [-0.278, -0.087] |
| Threat (not) exaggerated | -0.015 | 0.073 | [-0.160, 0.133] |
| Perceived COVID risk | 0.013 | 0.024 | [-0.026, 0.073] |

^†^Denotes a 95% bias-corrected bootstrap confidence interval (using 10,000 bootstrap samples) that does not include zero

*Supplementary Figure B.6. Direct effects of disgust sensitivity on COVID-19 test results mediated by COVID-specific predictors*

COVID knowledge

Disgust densitivity

Positive COVID-19 test

Threat (not) exaggerated

Perceived COVID risk

-0.316***

-0.082

0.271***

-0.958***

-0.006

1.188***

-0.128

**p* < 0.05; ***p* < 0.01; ****p* < 0.001

*Supplementary Table B.6. Indirect effects of disgust sensitivity on COVID-19 test results*

|  | Indirect effect | Standard error | 95% confidence interval |
| --- | --- | --- | --- |
| TOTAL | 0.628^†^ | 0.146 | [0.425, 1.010] |
| COVID knowledge | 0.306^†^ | 0.076 | [0.193, 0.494] |
| Threat (not) exaggerated | 0.001 | 0.026 | [-0.055, 0.057] |
| Perceived COVID risk | 0.321^†^ | 0.122 | [0.145, 0.622] |

^†^Denotes a 95% bias-corrected bootstrap confidence interval (using 10,000 bootstrap samples) that does not include zero

*Supplementary Figure B.7. Direct effects of perceived vulnerability to disease on COVID-19 test results mediated by COVID-specific predictors*

COVID knowledge

Perceived vulnerability to disease

Positive COVID-19 test

Threat (not) exaggerated

Perceived COVID risk

0.030

0.196***

0.419***

-0.954***

0.009

1.105***

0.108

**p* < 0.05; ***p* < 0.01; ****p* < 0.001

*Supplementary Table B.7. Indirect effects of perceived vulnerability to disease on COVID-19 test results*

|  | Indirect effect | Standard error | 95% confidence interval |
| --- | --- | --- | --- |
| TOTAL | 0.437^†^ | 0.180 | [0.163, 0.871] |
| COVID knowledge | -0.028 | 0.028 | [-0.087, 0.023] |
| Threat (not) exaggerated | 0.002 | 0.055 | [-0.108, 0.116] |
| Perceived COVID risk | 0.464^†^ | 0.170 | [0.213, 0.874] |

^†^Denotes a 95% bias-corrected bootstrap confidence interval (using 10,000 bootstrap samples) that does not include zero

*Supplementary Figure B.8. Direct effects of preexisting conditions on COVID-19 test results mediated by COVID-specific predictors*

COVID knowledge

Preexisting conditions

Positive COVID-19 test

Threat (not) exaggerated

Perceived COVID risk

-0.203***

0.051*

0.364***

-0.813***

-0.192

0.789***

0.034

**p* < 0.05; ***p* < 0.01; ****p* < 0.001

*Supplementary Table B.8. Indirect effects of preexisting conditions on COVID-19 test results*

|  | Indirect effect | Standard error | 95% confidence interval |
| --- | --- | --- | --- |
| TOTAL | 0.443^†^ | 0.074 | [0.317, 0.605] |
| COVID knowledge | 0.165^†^ | 0.028 | [0.115, 0.227] |
| Threat (not) exaggerated | -0.010 | 0.010 | [-0.032, 0.006] |
| Perceived COVID risk | 0.287^†^ | 0.070 | [0.163, 0.438] |

^†^Denotes a 95% bias-corrected bootstrap confidence interval (using 10,000 bootstrap samples) that does not include zero

*Supplementary Figure B.9. Direct effects of political ideology on COVID-19 test results mediated by COVID-specific predictors*

COVID knowledge

Political ideology^a^

Positive COVID-19 test

Threat (not) exaggerated

Perceived COVID risk

-0.263***

-0.413***

-135***

-0.849***

-0.211

0.783***

-0.196

^a^ Scale from 1 (extremely liberal) to 7 (extremely conservative)

**p* < 0.05; ***p* < 0.01; ****p* < 0.001

*Supplementary Table B.9. Indirect effects of political ideology on COVID-19 test results*

|  | Indirect effect | Standard error | 95% confidence interval |
| --- | --- | --- | --- |
| TOTAL | 0.205^†^ | 0.067 | [0.072, 0.336] |
| COVID knowledge | 0.224^†^ | 0.034 | [0.162, 0.298] |
| Threat (not) exaggerated | 0.087 | 0.068 | [-0.048, 0.221] |
| Perceived COVID risk | -0.106 ^†^ | 0.032 | [-0.178, -0.054] |

^†^Denotes a 95% bias-corrected bootstrap confidence interval (using 10,000 bootstrap samples) that does not include zero

*Supplementary Figure B.10. Direct effects of conspiratorial ideation on COVID-19 test results mediated by COVID-specific predictors*

COVID knowledge

Conspiratorial ideation

Positive COVID-19 test

Threat (not) exaggerated

Perceived COVID risk

-0.509***

-0.265***

0.102*

-0.693**

-0.479

1.004**

0.363

**p* < 0.05; ***p* < 0.01; ****p* < 0.001

*Supplementary Table B.10. Indirect effects of conspiratorial ideation on COVID-19 test results*

|  | Indirect effect | Standard error | 95% confidence interval |
| --- | --- | --- | --- |
| TOTAL | 0.582^†^ | 0.143 | [0.366, 0.932] |
| COVID knowledge | 0.353^†^ | 0.133 | [0.132, 0.665] |
| Threat (not) exaggerated | 0.127 | 0.099 | [-0.057, 0.339] |
| Perceived COVID risk | 0.102^†^ | 0.063 | [0.009, 0.254] |

^†^Denotes a 95% bias-corrected bootstrap confidence interval (using 10,000 bootstrap samples) that does not include zero

*Supplementary Figure B.11. Direct effects of science literacy on COVID-19 test results mediated by COVID-specific predictors*

COVID knowledge

Science literacy

Positive COVID-19 test

Threat (not) exaggerated

Perceived COVID risk

0.434***

0.159**

-0.121*

-0.715

-0.461

1.755*

0.753

**p* < 0.05; ***p* < 0.01; ****p* < 0.001

Note: The science literacy scale was included only in Study 1 at Time 1. Of the 304 participants who were responded to this measure, only 5 had positive COVID-19 tests. The bootstrapped mediational model failed to converge. Hence, the estimates should be interpreted cautiously.

*Supplementary Table B.11. Indirect effects of science literacy on COVID-19 test results*

|  | Indirect effect | Standard error | 95% confidence interval |
| --- | --- | --- | --- |
| TOTAL | -0.595^†^ | 295,828.911 | [-413.573, -0.186] |
| COVID knowledge | -0.310 | 111,285.505 | [-136.126, 0.322] |
| Threat (not) exaggerated | -0.073 | 88,852.714 | [-104.762, 0.361] |
| Perceived COVID risk | -0.212 | 122,323.743 | [-166.759, 0.019] |

^†^Denotes a 95% bias-corrected bootstrap confidence interval (using 10,000 bootstrap samples) that does not include zero

Note: The science literacy scale was included only in Study 1 at Time 1. Of the 304 participants who were administered this measure, only 5 had positive COVID-19 tests. The bootstrapped mediational model failed to converge. Hence, the estimates should be interpreted cautiously.

*Supplementary Figure B.12. Direct effects of Fox News use on COVID-19 test results mediated by COVID-specific predictors*

COVID knowledge

Fox News

Positive COVID-19 test

Threat (not) exaggerated

Perceived COVID risk

-0.273***

-0.364***

-0.030

-1.037***

-0.314

0.924***

-0.186

**p* < 0.05; ***p* < 0.01; ****p* < 0.001

*Supplementary Table B.12. Indirect effects of Fox News use on COVID-19 test results*

|  | Indirect effect | Standard error | 95% confidence interval |
| --- | --- | --- | --- |
| TOTAL | 0.370^†^ | 0.099 | [0.189, 0.580] |
| COVID knowledge | 0.284^†^ | 0.065 | [0.178, 0.433] |
| Threat (not) exaggerated | 0.114 | 0.089 | [-0.059, 0.298] |
| Perceived COVID risk | -0.028 | 0.046 | [-0.123, 0.057] |

^†^Denotes a 95% bias-corrected bootstrap confidence interval (using 10,000 bootstrap samples) that does not include zero

*Supplementary Figure B.13. Direct effects of NPR use on COVID-19 test results mediated by COVID-specific predictors*

COVID knowledge

NPR

Positive COVID-19 test

Threat (not) exaggerated

Perceived COVID risk

0.352***

0.350***

0.138**

-988***

-0.301

0.967***

-1.192

**p* < 0.05; ***p* < 0.01; ****p* < 0.001

*Supplementary Table B.13. Indirect effects of NPR use on COVID-19 test results*

|  | Indirect effect | Standard error | 95% confidence interval |
| --- | --- | --- | --- |
| TOTAL | -0.320^†^ | 0.102 | [-0.528, -0.126] |
| COVID knowledge | -0.348^†^ | 0.067 | [-0.500, -0.238] |
| Threat (not) exaggerated | -0.106 | 0.084 | [-0.283, 0.050] |
| Perceived COVID risk | -0.134^†^ | 0.064 | [0.033, 0.285] |

^†^Denotes a 95% bias-corrected bootstrap confidence interval (using 10,000 bootstrap samples) that does not include zero

*Supplementary Figure B.14. Direct effects of national newspaper and/or magazine use on COVID-19 test results mediated by COVID-specific predictors*

COVID knowledge

Newspapers and magazines

Positive COVID-19 test

Threat (not) exaggerated

Perceived COVID risk

0.213***

0.210***

0.089*

1.022***

-0.291

0.933***

-0.445

**p* < 0.05; ***p* < 0.01; ****p* < 0.001

*Supplementary Table B.14. Indirect effects of national newspaper and/or magazine use on COVID-19 test results*

|  | Indirect effect | Standard error | 95% confidence interval |
| --- | --- | --- | --- |
| TOTAL | -0.196^†^ | 0.077 | [-0.356, -0.050] |
| COVID knowledge | -0.218^†^ | 0.052 | [-0.338, -0.132] |
| Threat (not) exaggerated | -0.061 | 0.051 | [-0.168, 0.035] |
| Perceived COVID risk | 0.083^†^ | 0.044 | [0.010, 0.185] |

^†^Denotes a 95% bias-corrected bootstrap confidence interval (using 10,000 bootstrap samples) that does not include zero

*Supplementary Figure B.15. Direct effects of age on COVID-19 test results mediated by COVID-specific predictors*

COVID knowledge

Age

Positive COVID-19 test

Threat (not) exaggerated

Perceived COVID risk

0.073***

0.006

-0.025

-0.700***

-0.253

0.868***

-0.612

**p* < 0.05; ***p* < 0.01; ****p* < 0.001

*Supplementary Table B.15. Indirect effects of age on COVID-19 test results*

|  | Indirect effect | Standard error | 95% confidence interval |
| --- | --- | --- | --- |
| TOTAL | -0.074^†^ | 0.043 | [-0.176, -0.019] |
| COVID knowledge | -0.051^†^ | 0.028 | [-0.117, -0.016] |
| Threat (not) exaggerated | -0.002 | 0.007 | [-0.022, 0.009] |
| Perceived COVID risk | -0.022 | 0.020 | [-0.073, 0.004] |

^†^Denotes a 95% bias-corrected bootstrap confidence interval (using 10,000 bootstrap samples) that does not include zero

*Supplementary Figure B.16. Direct effects of gender on COVID-19 test results mediated by COVID-specific predictors*

COVID knowledge

Gender^a^

Positive COVID-19 test

Threat (not) exaggerated

Perceived COVID risk

-0.246 ***

-0.207***

-0.125**

-0.802***

-0.173

0.822***

0.250

^a^ Coded 1=male and 0=female; “Other” and “Prefer not to answer” responses were coded as missing for ease of interpretation

**p* < 0.05; ***p* < 0.01; ****p* < 0.001

*Supplementary Table B.16. Indirect effects of gender on COVID-19 test results*

|  | Indirect effect | Standard error | 95% confidence interval |
| --- | --- | --- | --- |
| TOTAL | -0.131 | 0.068 | [-0.005, 0.262] |
| COVID knowledge | 0.198^†^ | 0.043 | [0.122, 0.289] |
| Threat (not) exaggerated | 0.036 | 0.036 | [-0.034, 0.110] |
| Perceived COVID risk | -0.102^†^ | 0.044 | [-0.204, -0.029] |

^†^Denotes a 95% bias-corrected bootstrap confidence interval (using 10,000 bootstrap samples) that does not include zero

*Supplementary Figure B.17. Direct effects of race on COVID-19 test results mediated by COVID-specific predictors*

COVID knowledge

Race^a^

Positive COVID-19 test

Threat (not) exaggerated

Perceived COVID risk

-1.052***

-0.069

0.355***

-0.871***

-0.148

0.696***

-0.011

^a^ Coded 1=Black and 0=white; all other racial and ethnic categories were coded as missing for ease of interpretation

**p* < 0.05; ***p* < 0.01; ****p* < 0.001

*Supplementary Table B.17. Indirect effects of race on COVID-19 test results*

|  | Indirect effect | Standard error | 95% confidence interval |
| --- | --- | --- | --- |
| TOTAL | 1.173^†^ | 0.184 | [0.849, 1.563] |
| COVID knowledge | 0.916^†^ | 0.154 | [0.648, 1.257] |
| Threat (not) exaggerated | 0.010 | 0.021 | [-0.025, 0.062] |
| Perceived COVID risk | 0.247^†^ | 0.090 | [0.103, 0.455] |

^†^Denotes a 95% bias-corrected bootstrap confidence interval (using 10,000 bootstrap samples) that does not include zero
